# Supplementary material for: Cloud BioLinux: pre-configured and on-demand bioinformatics computing for the genomics community
Source: BMC Bioinformatics. 2012 Mar 19;13:42. doi: 10.1186/1471-2105-13-42 (PMC3372431; doi:10.1186/1471-2105-13-42)
Supplement: Additional file 1 — Supplementary 1 Cloud BioLinux software documentation in the form of a mini, self-contained website. Users need to download and uncompress the .zip file, and open through a web browser the "index.html" file available on the main directory. (ZIP 1823 kb). [file 1471-2105-13-42-S1.ZIP › Cloud-BioLinux-Package-Documentation/docs/gcphrap.html]

Bio-Linux Software Documentation Pages

Back to search form

## gcphrap

|  |  |
| --- | --- |
| Name | gcphrap |
| Description | **gcphrap** is a modified version of the phrap progam which allows easy integration with the pregap 4 and gap4 (staden package) processing, editing and finishing tools. This includes providing a graphical user interface from within pregap4 and gap4 for running phrap, and also additions to the display and search functions in the gap4 contig editor to make further use of confidence values generated by phred. For phrap and gap4 integration, you need to install gcphrap.  **gcphrap** itself is not licensed software, but requires the phrap package, which is. Please visit the official phrap web pages for details on how to obtain phrap.  Please read the official availability and installation information for gcphrap. |
| Homepage | http://staden.sourceforge.net/phrap.html |
| Remote Documentation | http://staden.sourceforge.net/phrap.html |
